# The rational search for selective anticancer derivatives of the peptide Trichogin GA IV: a multi-technique biophysical approach

**DOI:** 10.1038/srep24000

**Published:** 2016-04-04

**Authors:** Annalisa Dalzini, Christian Bergamini, Barbara Biondi, Marta De Zotti, Giacomo Panighel, Romana Fato, Cristina Peggion, Marco Bortolus, Anna Lisa Maniero

**Affiliations:** 1 Dipartimento di Chimica, Università di Padova, via Marzolo 1, 35131, Padova, Italy; 2 Dipartimento di Farmacia e Biotecnologie, Università di Bologna, via Irnerio 48, 40126, Bologna, Italy; 3 Dipartimento di Scienza dei Materiali, Università degli Studi di Milano Bicocca, 20126, Milano, Italy

## Abstract

Peptaibols are peculiar peptides produced by fungi as weapons against other microorganisms. Previous studies showed that peptaibols are promising peptide-based drugs because they act against cell membranes rather than a specific target, thus lowering the possibility of the onset of multi-drug resistance, and they possess non-coded α-amino acid residues that confer proteolytic resistance. Trichogin GA IV (TG) is a short peptaibol displaying antimicrobial and cytotoxic activity. In the present work, we studied thirteen TG analogues, adopting a multidisciplinary approach. We showed that the cytotoxicity is tuneable by single amino-acids substitutions. Many analogues maintain the same level of non-selective cytotoxicity of TG and three analogues are completely non-toxic. Two promising lead compounds, characterized by the introduction of a positively charged unnatural amino-acid in the hydrophobic face of the helix, selectively kill T67 cancer cells without affecting healthy cells. To explain the determinants of the cytotoxicity, we investigated the structural parameters of the peptides, their cell-binding properties, cell localization, and dynamics in the membrane, as well as the cell membrane composition. We show that, while cytotoxicity is governed by the fine balance between the amphipathicity and hydrophobicity, the selectivity depends also on the expression of negatively charged phospholipids on the cell surface.

Antimicrobial peptides (AMPs) attack bacterial cells by disrupting the plasma membrane without binding to a specific receptor, thus circumventing the ability of the cells to adapt and develop drug resistance. Some AMPs have been proposed also as antitumor molecules. Indeed, they display a selective cytotoxicity towards cancer cells ascribed to the different composition and physical properties of the cancer cell membrane relative to healthy cells^1^,^2^,^3^,^4^. Besides the differences in the composition of lipid side chains, the external surface of cancer cell membranes typically carries a net negative charge due to an elevated expression of anionic molecules, such as phosphatidylserine (PS)^5^,^6^. In contrast, the surface of healthy mammalian cell membranes is mainly composed of zwitterionic phospholipids and sterols, such as cholesterol. It has also been suggested that cholesterol, altering membrane fluidity, may interfere with AMPs activity^7^: since cancer cells show modified levels of cholesterol, this may be a further reason for the selectivity displayed by some AMPs. The sequences of AMPs explored so far are extremely variable, but the large majority have common characteristics: they are positively charged and adopt an amphiphilic helical structure in the membrane, sometimes interrupted by a flexible central segment that contains glycine or proline residues. The large amount of data collected on AMPs indicates that their strength and mechanisms of interaction with membranes strongly depend on the combination of several features including main-chain length, helical secondary structure, charge, hydrophobicity and amphipathic character^8^,^9^. While promising, AMPs have several shortcomings that need to be addressed before they can be considered as chemotherapeutic drugs, among them a low stability in the serum, leading to poor pharmacokinetic properties. Additionally, the lack of a univocal correlation between the chemical and structural properties of AMPs and their biological activity impairs the rational synthesis of improved molecules.

In this context, we focus on peptaibols, a class of hydrophobic AMPs characterized by the presence of an acyl group at the N-terminus and an alcoholic function at the C-terminus. Moreover, peptaibols sequences exhibit an abundance of non-coded α-amino acids^10^, the most recurring being the C^α^-tetrasubstituted α-aminoisobutyric acid (Aib, Fig. 1), known to confer to peptaibols a significant resistance to enzyme hydrolysis^11^ and to promote the formation of a helical secondary structure even in short sequences. Trichogin GA IV (TG) is a short (10 residues long) peptaibol, produced by the fungus *Trichoderma longibrachiatum*; its primary structure is: 1-Oct-Aib-Gly-Leu-Aib-Gly-Gly-Leu-Aib-Gly-Ile-Lol^12^,^13^. 1-Oct is the fatty acid 1-Octanoyl, and Lol is the amino alcohol leucinol: both play a crucial role in the antimicrobial and cytotoxic activity of this peptide. For the N-terminus, it was discovered that at least six carbon atoms are required for toxicity, but too long a chain results in activity loss as well^14^. As regards the C-terminus, a recent study demonstrated that the replacement of the amino-alcohol with a methyl ester or a carboxylic acid determines a significant inhibition of the cytotoxic action of TG in cells^15^. TG adopts a helical structure in methanol and in micelles of sodium dodecyl sulphate (SDS)^16^. TG is a very versatile scaffold: each of its analogues, even carrying only one variation in the amino acid sequence, possesses peculiar biological features, such as selectivity against bacterial or fungal strains or low haemolytic activity^11^. Moreover, being that TG is only 10 residues long, the synthetic effort is reduced thus enabling a fast synthesis of promising analogues.

In this work, we designed and synthesized thirteen TG analogues having different charge, hydrophobicity and amphipathicity. We chose both natural (Arg, Lys, Leu) and unnatural amino acids (Api, 4-aminopiperidine-4-carboxylic acid, Fig. 1)^17^,^18^ as substituents in different sectors of the peptide to modulate the key properties listed above. The sequences of the analogues are reported in Table 1, along with structural and biological properties. We determined the secondary structure of the analogues in MeOH, in SDS and in small unilamellar vesicles (SUV) composed of POPC (1-palmitoyl-2-oleoyl-*sn*-glycero-3-phosphocholine) using circular dichroism (CD) spectroscopy. Their hydrophobicity and amphipathicity were quantified by means of the widely used CCS scale^19^,^20^, extended to include the peculiar residues that characterize TG and its analogues. We evaluated the cytotoxic activity of the peptides using the MTT assay, testing them against three cell lines: a primary line of human dermal fibroblasts (HDF), a cell line derived from human glioma cells (T67) and the classic line derived from cervical cancer cells (HeLa). The total amount of cholesterol and the level of PS expression on the outer leaflet of the plasma membrane of the three cell lines were quantified with biochemical assays. The C^α^-tetrasubstituted spin label amino acid TOAC (2,2,6,6-tetramethylpiperidine-1-oxyl-4-amino-4-carboxylic acid, Fig. 1) was inserted in several analogues to study their membrane binding properties in SUV and in cells using electron paramagnetic resonance (EPR) spectroscopy. Finally, fluorescence microscopy studies were performed on two additional analogues containing the fluorescent probe fluorescein isothiocyanate (FITC) interacting with HeLa cells to study the peptide cellular localization.

## Results

### Design strategy

**Figure 1:**
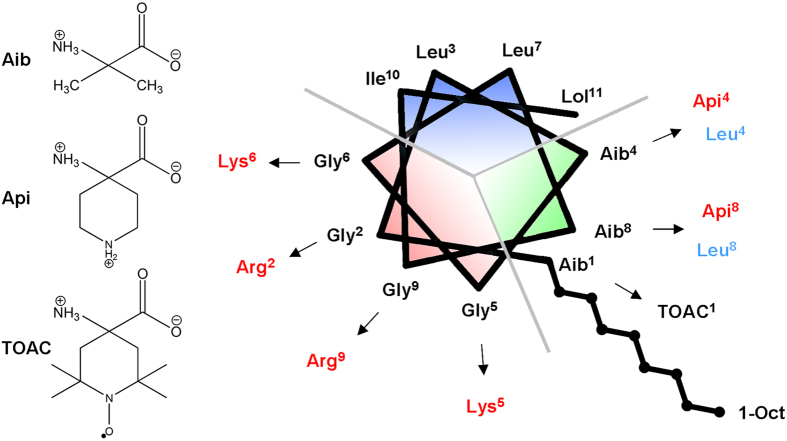
On the left, the structure of the three unusual amino-acids used in this work. On the right, helical wheel representation of TG. The octanoyl tail present at the N-terminus is shown in the bottom right part. The arrows indicate the substitutions: charged substitutions are in red, hydrophobic substitutions in blue. The three grey lines divide the peptide in three sectors (see text).

The sequence of TG is shown in the helical wheel representation in Fig. 1. TG is a hydrophobic peptide with a slightly amphipathic character. Indeed, as found for a large number of amphipathic α-helical AMPs^19^, its residues are spatially distributed so one face of the helix is occupied by slightly hydrophilic residues, while the other face is strongly hydrophobic. As shown in Fig. 1, the helical wheel can be divided into three sectors: a slightly hydrophilic sector of ~140°, entirely made of Gly residues (red), a highly hydrophobic sector of ~130° (blue), and a sector of ~90° occupied by the three Aib residues that, being helix-promoting, have a structural function.

We designed and synthesized various analogues of TG, in which the substitutions, highlighted in Fig. 1, were chosen to modify charge, hydrophobicity and amphipathicity of the natural peptide. Since a broad hydrophobic core is a prerequisite for effective membrane perturbation^21^, we did not perturb the blue sector of TG, instead focusing our attention on the hydrophilic (red) and structural (green) sectors. In order to enhance the mild amphipathic character of TG, we introduced the positively charged Lys or Arg in place of Gly. The enhancement of the overall hydrophobicity was achieved by Aib-to-Leu substitutions. To perturb the amphipathicity of the helix we chose Aib-to-Api substitutions. The sequences of all analogues are reported in Table 1.

Lys or Arg residues were introduced in the sequence in the positions naturally occupied by Gly. These substitutions should not preclude helical formation, but aim at introducing charges on the hydrophilic side of the helix. The occurrence of these two amino acids in natural peptides and proteins is dependent on the particular biological function^22^, and arginine-rich peptides are known for their cell-penetrating properties^23^. The antibacterial activity of [Lys^6^] TG and [Lys^5,6^] TG have already been characterized and [Lys^5,6^] TG showed a remarkably increased activity against some bacterial strains, in particular against *S. aureus*^24^. Here, TG analogues containing either Lys or Arg have been prepared to assess whether the type of charged residue influences the toxicity of the peptide towards the cells.

Leu is a known helix-supporting residue, although less effective than Aib, so Leu in position 4 or 8 should not heavily perturb the helicity and stability of the helix. Previous studies demonstrated that [Leu^8^] TG has an improved antibacterial activity against *S. aureus* and a good proteolytic stability, while Leu in position 4 brings to a loss of antibacterial activity^7^.

Perturbation of the hydrophobic/hydrophilic balance in TG has been achieved by inserting an Api residue. This substitution was chosen since it has been previously reported that a slight perturbation of amphipathicity, *via* the introduction of a charged residue in the hydrophobic sector, leads to an improvement in the selectivity of peptides^25^. Api, a positively charged C^α^-tetrasubstituted amino acid, has been placed in positions naturally occupied by Aib, so to guarantee the maintenance of helicity in TG analogues.

In several analogues we introduced either a paramagnetic or a fluorescent label to study the peptide binding and localization into the cells. The paramagnetic analogues carry a TOAC residue at position 1, a place normally occupied by an Aib residue. TOAC is of primary importance in the study of the Aib-rich family of peptaibols, since it shares with Aib the characteristic of being a conformationally constrained C^α^–tetrasubstituted amino acid; they both are strong helix inducers and many peptaibols have been modified substituting TOAC in places of the primary sequence occupied by an Aib residue^26^,^27^. The fluorescent derivatives were obtained labelling peptides with fluorescein isothiocyanate (FITC), a derivative of fluorescein widely used as a fluorescent tracer in proteins. We synthesized two analogues with the label placed at the C-terminal: the final Lol residue has been replaced with a Leu to which a FITC has been linked *via* ester formation. One of the analogues has the natural TG sequence, while the other has a Gly-to-Lys substitution at position 6 (Oct-UGLUGGLUGIL-FITC and Oct-UGLUG**K**LUGIL- FITC).

### Synthesis

All trichogin GA IV analogues were prepared by automatic Solid Phase Peptide Synthesis (SPPS) applying our improved protocol for the preparation of peptaibols^24^,^26^. The syntheses were performed on the L-Lol substituted 2-chlorotrityl resin^27^,^28^,^29^ as described in detail in the Methods section. Our procedure involves the use of the strong activating method *via* HATU or EDC/HOAt in the coupling reactions with Aib and TOAC residues, and cleavage from the 2-chlorotrityl resin^29^ in three cycles of 30% HFIP in distilled CH_2_Cl_2_ (the last cycle performed overnight). When Lys or Arg are in the sequence, a removal protocol with strong acid (TFA or HCl) was required. In this case, for TOAC containing peptides, a further treatment with ammonia is needed to regenerate the nitroxyl group, that can be partially damaged by the acidic treatment performed for the cleavage of the peptide from the resin^30^. The desired final peptides were isolated in 75–80% average yield and purified by reverse-phase liquid chromatography to an average of 98% purity. The peptides were additionally characterized by ESI–MS and NMR spectrometry.

### Cytotoxicity assay

We studied the effect of TG and its analogues on normal (HDF) and transformed (T67 and HeLa) cells. The cellular viability was assessed after 24 hours of exposure to the peptides by the MTT assay (Table 1).

The sequence of the main isoform of trichogin GA IV (TG) showed moderate toxicity with EC50 values of 2, 4 and 8 μM for T67, HeLa and HDF, respectively. Overall, the enhancement of the amphipathic character of the peptide with the introduction of charged residues in the hydrophilic face does not improve the toxicity. The introduction of a single Lys at position 6 kept the toxicity intact, while the introduction of two Lys, at positions 5 and 6, or of a single Arg, in positions 2 or 9, slightly lowered it. The substitutions in the structural/hydrophobic face of the peptide, introducing a Leu in place of an Aib in position 4 or 8, had a more dramatic effect, completely abolishing the cytotoxic activity of the peptides up to 20 μM. From the point of view of selectivity, the best results were obtained with the introduction of the charged Api amino acid at position 4 or 8. Api conferred to the TG sequence a selective toxicity against T67 cells, in fact no cytotoxic effects were observed on the other two lines. The analogues labelled with FITC could not be reliably tested on cell viability since the label interferes with the MTT assay.

In light of the MTT assay results, we decided to focus our attention for the spectroscopic studies on four peptides that are representative of the different behaviours displayed by the peptides towards the cells: [TOAC^1^] TG as the reference peptide; [TOAC^1^, Leu^4^] TG that shows no toxicity; [TOAC^1^, Lys^6^] TG that is the most toxic peptide on all the cell lines; [TOAC^1^, Api^4^] TG that has a selective toxicity. We chose to use the analogues bearing the TOAC residue at position 1 for both CD and EPR experiments since the insertion of the spin labelled amino acid TOAC at position 1 did not significantly influence the cytotoxicity in any of the analogues, as previously observed for the bioactivity of other peptides^31^. These results confirm that the TOAC label is an optimal choice for biological studies, not only for EPR, but also for those techniques that rely on a paramagnetic probe as a quencher, such as fluorescence or NMR^32^,^33^.

### Cell membrane constituents assays

**Figure 2:**
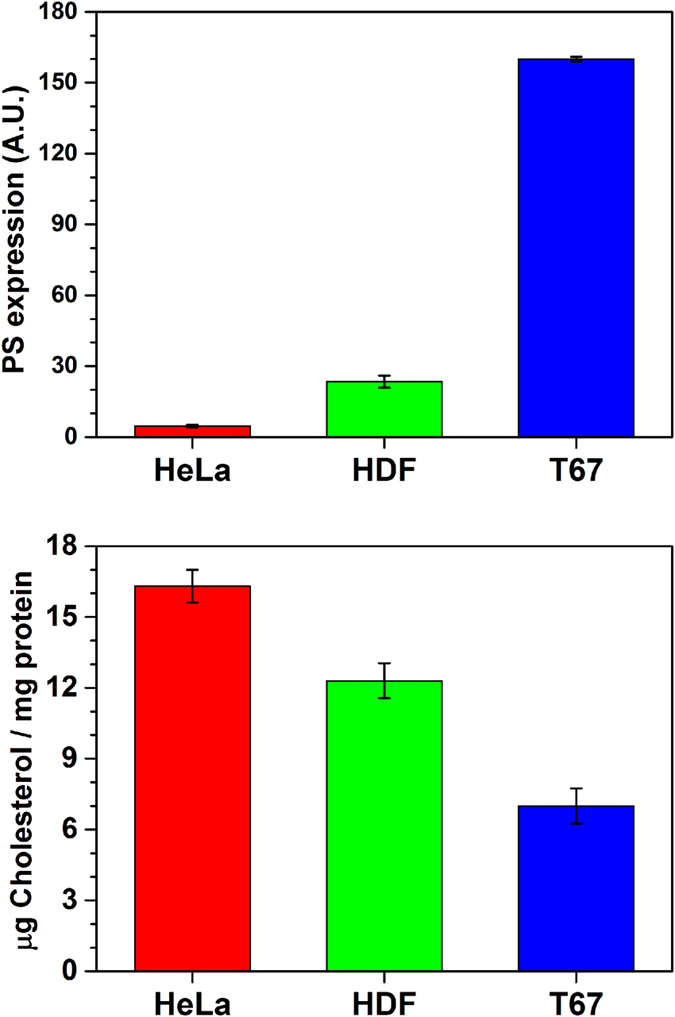
Top, Annexin V assay to quantify the amount of PS exposed on the outer cell membrane. Bottom, cholesterol content assay, quantifying the total cellular cholesterol. The error bars are relative to the standard deviation of three repeated experiments. The quantification of the cellular levels of PS in living cells was performed by flow cytometry using Alexa Fluor^®^ 488 Annexin V Kit (Molecular Probes). Total cellular cholesterol was quantified by reverse phase HPLC on cellular homogenates previously treated with cholesterol oxidase and cholesterol esterase.

We tested the different cell lines for the exposition of PS on the membrane surface and for their total amount of cholesterol (Fig. 2). It has to be noted that ~90% of free cholesterol was estimated to reside in the plasma membrane in several cell types, including human fibroblasts^34^,^35^. The details of both assays are reported in the Methods section.

The amount of PS and cholesterol in the plasma membrane has been suggested to partake in the selectivity of AMPs towards tumour cells^3^. The negative charge favours the binding of positively charged peptides on the membrane surface, while cholesterol, altering the membrane fluidity, may interfere with the peptide binding. The three cell lines are characterized by significantly different compositions of the outer surface of the plasma membrane. T67 cells have high negative surface charge (high PS expression on the outer membrane leaflet) and low cholesterol levels, a composition that is typical of tumour cells. HDF have a composition that is expected for a healthy primary cell line, i.e. low negative surface charge and high cholesterol. HeLa, have an unusual profile for a cancer cell line, more similar to normal cells: it has the highest cholesterol levels and lowest negative surface charge of the three lines we studied.

### CD experiments in model membranes

Peptides have been studied by far-UV CD in MeOH and in SDS micellar environment and SUV of POPC to assess their secondary structure. For all the analogues, in the CD spectra we can observe two negative Cotton effects at 204–206 nm and 220–224 nm and a positive Cotton effect at about 195 nm, features typical of right-handed helix conformations. The first two negative maxima are related to the parallel component of the π → π* transition and to the n → π* transition of the peptide chromophore, while the positive maximum is related to the perpendicular component of the π → π* transition^36^. An estimation of the helical type (whether 3_10_ or α) can be achieved by the ratio of the two negative maxima^14^,^36^,^37^ of the ellipticity, R = [θ]_222_/[θ]_208_, reported in Table 1. In the spectra registered in MeOH this parameter ranges from 0.2 to 0.6, indicating that the peptides adopt mostly a well-established 3_10_-helix. In SDS solution, the experimental R value increases for almost all of them, ranging from 0.4 to 0.8. These values are consistent with the presence of a prevalent α-helical conformation. Therefore, a large change in R upon changing the solvent indicates that the structure of the peptide is able to flip from 3_10_ to α helix.

**Figure 3:**
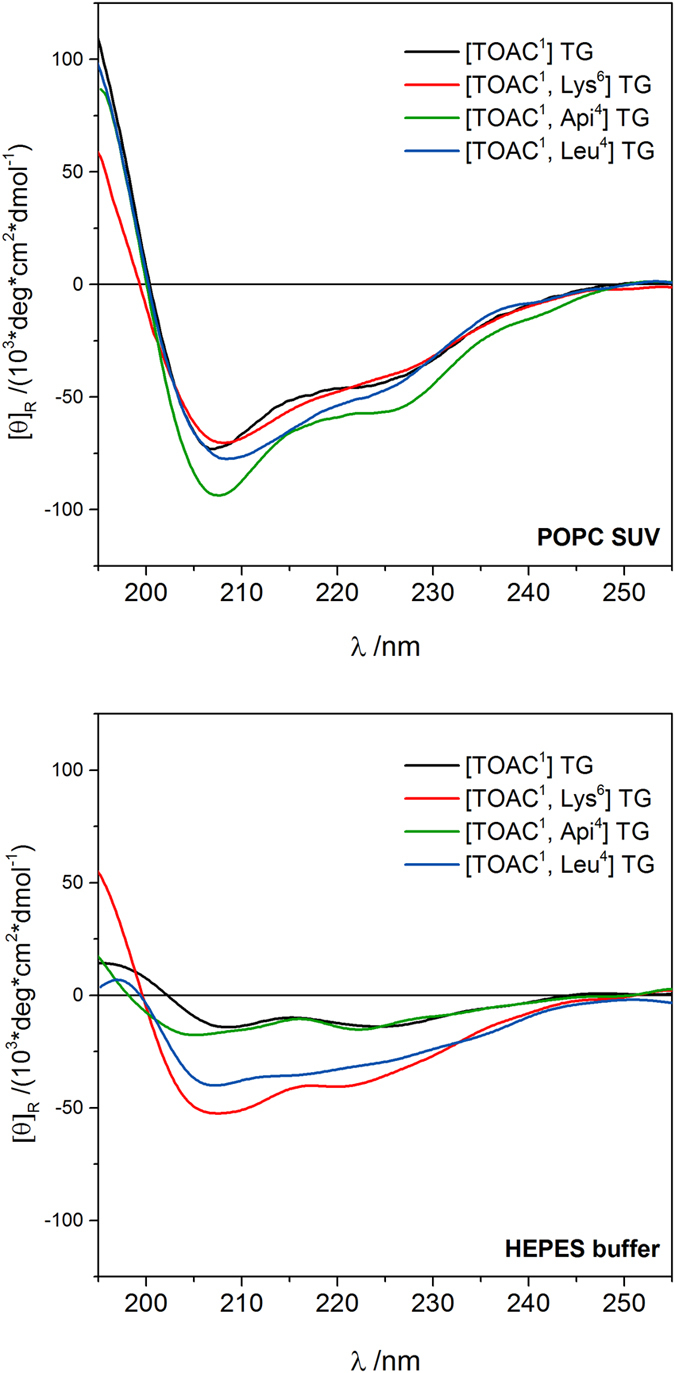
Far-UV/CD spectra of [TOAC^1^] TG, [TOAC^1^, Api^4^] TG, [TOAC^1^, Lys^6^] TG and [TOAC^1^, Leu^4^] TG in 2 mM POPC SUV (top) and in buffer solution (bottom). Peptide concentration, 0.1 mM.

SDS is useful as a model lipid system for a rapid screening of peptide structure in a membrane-like environment, but the structure adopted in micelles may not fully reflect the one in bilayer membranes. Therefore, we recorded the CD spectra of the four selected analogues (see *cytotoxicity assay* paragraph) in a better membrane-mimicking system, i.e. SUV composed by POPC at a peptide:lipid ratio of 1:20 (Fig. 3, top). We used POPC SUV as a model membrane in the fluid phase, since normal eukaryotic plasma membranes are composed primarily by zwitterionic phospholipids. The shape of the CD spectra are all consistent with the presence of a well-developed, right-handed, mixed 3_10_/α-helical conformation in membrane (R ≈0.6). We can safely conclude that the peptide helical conformation is conserved in phospholipid bilayer.

Additionally, we recorded the CD spectra of the selected analogues in buffer solution (Fig. 3, bottom). The spectra show an overall decrease in the molar ellipticity for all analogues, indicating a generalized loss of secondary structure. [TOAC^1^, Lys^6^] TG and [TOAC^1^, Leu^4^] TG show only a minimal reduction of the ellipticity and conserve the same lineshape displayed in SUV. [TOAC^1^] TG and [TOAC^1^, Api^4^] TG conserve the two minima typical of the α-helix, but with extremely reduced ellipticity.

### Mean hydrophobicity and hydrophobic moment

Mean hydrophobicity (H) and mean hydrophobic moment (μ) are widely used to characterize peptide sequences^38^. H depends only on the primary sequence and is higher for more hydrophobic peptides, while μ depends on the peptide secondary structure and is directly proportional to peptides amphipathicity. The values of H and μ for all analogues are reported in Table 1.

Several scales have been developed to quantify amino-acids hydrophobicity. Among them, the new Combined Consensus Hydrophobicity Scale (CCS), developed by Tossi *et al.*^19^,^20^, was derived from a set of 162 theoretical and experimental scales and it defines the hydrophobicity of the amino acids in a scale from −10 (Arg, considered the most hydrophilic among standard amino-acids) to +10 (Phe, considered the most hydrophobic amino-acid). We chose to use the CCS scale as it comprises the hydrophobicity of some non proteinogenic amino-acids, in particular Aib that is present in all our TG analogues. However, the CCS scale does not include the amino acids Api and TOAC. Additionally, TG and its derivatives are characterized by peculiar N- and C-terminal groups (1-Oct and Lol, respectively) that are fundamental for the cytotoxic effect^15^. It is possible to calculate the H values for the missing groups exploiting the good correlation^19^,^20^ of the CCS scale with the octanol/water partition coefficient (logP) for N-acetylated and C-amidated residues. Therefore, we calculated logP as the average of the slightly different values obtained from different software (Kowwin v. 1.68 and ChemDraw v. 9.0) and extrapolated H values on the CCS scale. The obtained values are the following: Api = −6.9; TOAC = +0.8; Lol = +12.9; 1-Oct-Aib = +23.9; 1-Oct-TOAC = +23.3. Given the extremely high hydrophobicity of the alkyl chain, the residues that include the octanoyl chain at the N-terminus have H values that widely exceed the value +10 of Phe. From the individual H values, we calculated the mean hydrophobicity per residue as the mediated hydrophobicity of all the amino acids of a peptide sequence. The validity of the calculated H values has been confirmed by comparison with the retention times in reverse phase HPLC of selected peptides (Fig. S6).

The hydrophobic moment is defined as follows:


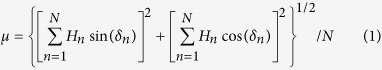


where H_n_ is the hydrophobicity of the amino acid n and δ_n_ is the angle at which successive side chains emerge from the backbone when the helix is viewed down its axis. Given the results of CD spectra in SUV (Fig. 3), we used an angle of 100°, typical of an α-helical structure, for all the peptides. Indeed, it was previously reported^39^ that the 3_10_ portion of the TG helix is confined to the first three residues at the N-terminus. Using an angle δ_n_ of 120° (typical of the 3_10_ helix) for amino acids from 1 to 3, the final value of μ does not vary significantly.

### EPR experiments in model and cells membranes

**Figure 4:**
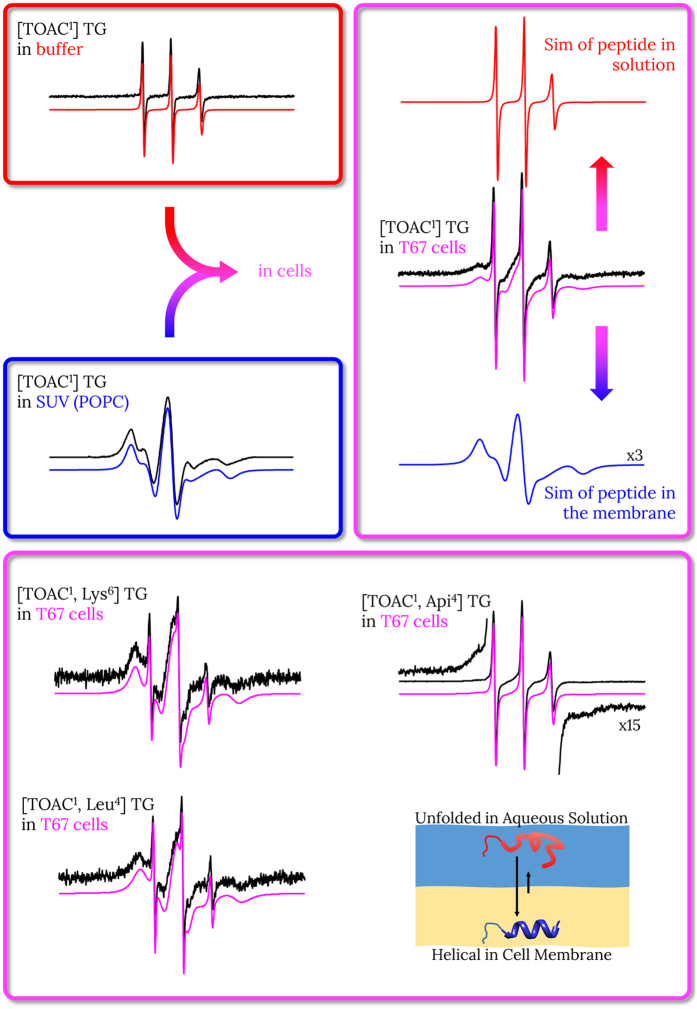
EPR spectra and simulations of TOAC-labelled peptides in buffer (red), in SUV (blue), and in suspension of T67 cells (magenta). In the top part of the figure, we show that the spectrum of [TOAC^1^] TG interacting with cells can be simulated as the sum of the peptide in aqueous solution and bound to the membrane (the salient parameters of the simulations are reported in Table 2). In the bottom part of the figure, we show the spectra and the simulations of the other three selected peptides interacting with T67 cells. The side wings of the spectrum of [TOAC^1^, Api^4^] TG are also shown enlarged to better highlight the membrane-bound component. The width of each spectrum is 14 mT. The cartoon shows the folding and the binding equilibrium of the peptide.

EPR spectroscopy has been performed on TOAC-labelled analogues to determine the amount of peptide bound to the membrane and to evaluate the peptide dynamics. The EPR spectrum of a nitroxide is very sensitive to its motional parameters, and peptides in phases of different viscosities experience different dynamics and exhibit characteristic lineshapes. We studied all analogues in buffer solution and in POPC SUV, the same systems used for CD spectroscopy; four selected analogues (see *cytotoxicity assay* paragraph) were also studied interacting with a suspension of intact cells of the three cell lines. Table 2 reports the results on the dynamics (quantitatively expressed by the isotropic rotational correlation time, τ) and the percentage of bound peptides: these data have been obtained from the simulation of the spectra. Additionally, from the simulations, we determined the value of the A_zz_ hyperfine component that gives an indication of the polarity of the environment. All EPR spectra and simulations are reported in the Supplementary Information (Figs S1–S4), while in Fig. 4, we report an overview of the experiments. In the top part of the figure, we show the spectra of [TOAC^1^] TG in buffer solution (blue), in SUV (red), and interacting with T67 cells (magenta). In the bottom part, we show the spectra of the other three peptides, representative of the different degrees of biological activity, interacting with intact T67 cells.

The EPR spectra of all analogues in buffer solution are identical to the spectrum in buffer reported in Fig. 4. The spectrum shows three sharp lines that are the fingerprint of a mostly unfolded peptide, as confirmed by the CD data. The sharp lines are caused by the relatively fast motion on the EPR timescale (rotational correlation time τ ≈ 0.2 ns) of the TOAC label when inserted in an unstructured peptide. The A_zz_ hyperfine component in the aqueous solution is 3.73 mT, a value that is compatible with a nitroxide in a highly polar aqueous environment.

The EPR spectra of all TOAC-labelled peptides recorded in POPC SUV are very similar to each other, and are shown in the Supplementary Information, Fig. S1. The spectra in SUV exhibit a broad lineshape indicative of a slow motion on the EPR timescale (τ ≈ 10 ns), characteristic of a peptide bound to the membrane. Then, we can conclude that all peptides are fully bound (>99%) to the POPC SUV. As expected for a membrane-bound peptide, the A_zz_ hyperfine component is much lower than in buffer, 3.27 mT: this value suggests that the TOAC label experiences an apolar environment compatible with the bilayer interior rather than the aqueous phase. Additionally, since all spectra have almost the same lineshape, there are no significant variations in the dynamics of the different analogues when bound to the membrane.

The EPR spectra of all peptides interacting with suspensions of intact cells (Fig. S3) show two spectral components: the three sharp lines characteristic of peptides in aqueous solution and the broad lineshape of peptides bound to the membrane. Therefore all peptides are not fully bound to the cell membranes. The presence of two well-defined spectral components implies that the exchange of the peptides between the two phases (membrane and aqueous solution) is slow on the EPR timescale, with a frequency less than ≈1 MHz. The amount of peptide bound to the membrane is obtained from the relative contribution of each spectral component to the total area of the simulated spectrum. A detailed example of the procedure is reported in the Supplementary Information, Fig. S4. The spectra show small variations in binding for the same peptide in different cell lines, and large variations for different peptides in the same cell line. Among the analogues, [TOAC^1^, Lys^6^] TG and [TOAC^1^, Leu^4^] TG show the largest percentages of bound peptide (≥90%), [TOAC^1^] TG is slightly less bound to the cells (~80%), while [TOAC^1^, Api^4^] TG is mostly free in solution (<40% bound). There are no significant differences in the dynamics of the membrane-bound peptides neither between different analogues, nor between different cell lines. Additionally, the dynamics is identical to those in POPC SUV, while the polarity of the environment is slightly higher in cells than in SUV with an A_zz_ hyperfine component of 3.35 mT for the cell-bound peptides.

### Fluorescence microscopy

**Figure 5:**
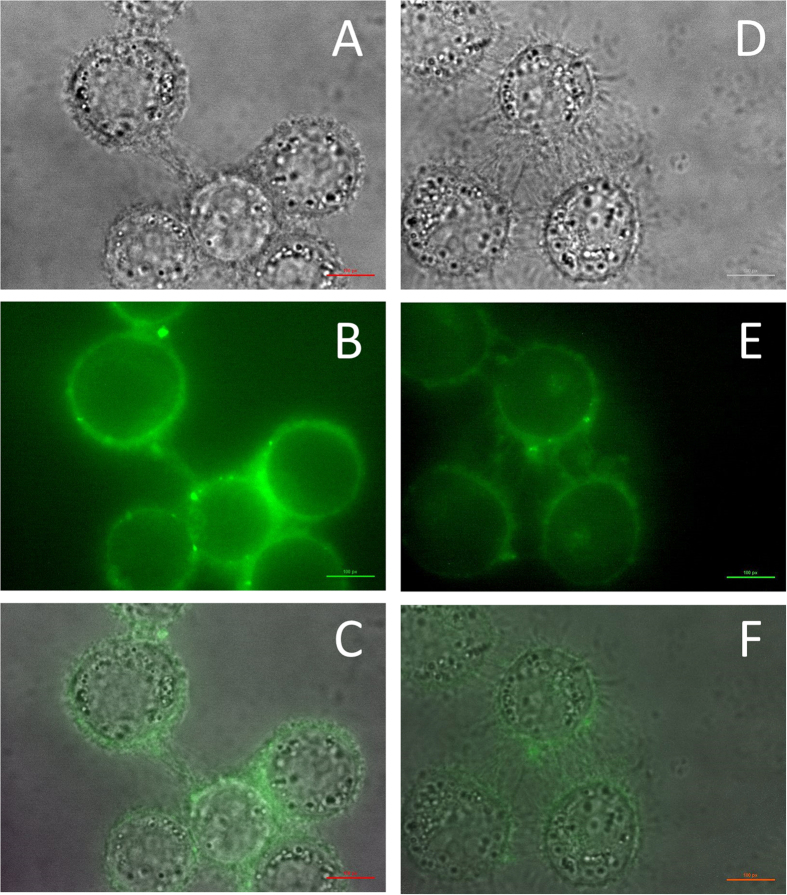
Fluorescence microscopy of HeLa cells treated with 0.5 μM of TG-FITC conjugate analogues for 3 hours. Panels (**A**,**D**) refer to the bright field images in the presence of [FITC^11^] TG and [Lys^6^, FITC^11^] TG respectively. Panels (**B**,**E**) refer to the green fluorescence images in the presence of [FITC^11^] TG and [Lys^6^, FITC^11^] TG respectively. Panels (**C**,**F**) refer to the merging of bright and fluorescence fields (see the Methods section for details).

HeLa cells were treated for 3 hours with 0.5 μM of TG and [Lys^6^] TG labelled in position 11 with the green fluorochrome FITC and the green signal (at 545 nm) was observed with a fluorescence microscope (Fig. 5). The micrographs reported in Fig. 5B,E clearly show that the fluorescent label is mainly located in the outer plasma membrane according to the hydrophobic nature of the TG analogues. The cells treated with [FITC^11^] TG are largely intact (Fig. 5A) as expected for a TG analogue lacking the C-terminal amino-alcohol that is essential for cytotoxicity^15^. Conversely, treatment with [Lys^6^, FITC^11^] TG induces severe membrane lysis, as shown by the membrane filaments appearing in-between the cells (Fig. 5D), suggesting that Lys in position 6 counterbalances the lack of the C-terminal amino-alcohol. It has been shown that some AMP act not on the plasma membrane, but on the mitochondria^3^. Therefore, we performed additional experiments staining the mitochondria with rhodamine (Fig. S5): the experiments do not show any colocalization of the peptides and rhodamine, confirming that TG analogues do not locate inside the mitochondria.

## Discussion

Our goal with the present study was to explore the determinants of the cytotoxicity displayed by trichogin GA IV and its analogues in order to guide further synthetic efforts towards selective peptides. In order to reach our goal, it is of paramount importance to consider both the cell killing mechanisms and the determinants of the selectivity. For the majority of AMPs, the main cytotoxic mechanism of action is the lysis of the cell membrane that follows the peptide binding. Both binding and lysis are governed by several parameters tied to the chemical nature of the peptides and their 3D-structure^19^,^21^,^40^,^41^. At the same time, the selectivity is chiefly governed by differences in cell membranes composition^2^,^4^. Therefore, to interpret the cytotoxicity of the TG analogues (Table 1), we followed three parallel routes: (1) we assessed the chemical/structural parameters of the peptides (Table 1 and Fig. 3); (2) we characterized a few key components of the cell membrane of the three chosen cell line (Fig. 2); (3) we studied the binding, localization and dynamics of the TG analogues interacting with the cells (Figs 4 and 5).

We varied the hydrophobicity (H), charge and hydrophobic moment (μ) of the natural sequence of TG (see Table 1) in the design of our analogues: CD data (Fig. 3) confirm that the helicity of the peptides, in membrane-like environments, is conserved in all analogues. The natural TG sequence displays a micromolar cytotoxicity towards both cancer and primary cell lines. None of the explored substitutions significantly increased the efficacy of natural TG. Unexpectedly, the introduction of a charge in the hydrophilic domain kept the cytotoxicity almost unaltered and the introduction of two charges slightly reduced it. On the other hand, increasing the overall hydrophobicity with the Aib-to-Leu substitutions completely abolished the peptide cytotoxicity. Intriguing is the effect of the perturbation of the helix amphipathic balance with the Aib-to-Api substitutions, that resulted in a selective toxicity of these peptides towards the T67 cell line; this behaviour supports a previous observation of an improved selectivity caused by the destabilization of the amphipathicity of the helix^25^.

**Figure 6:**
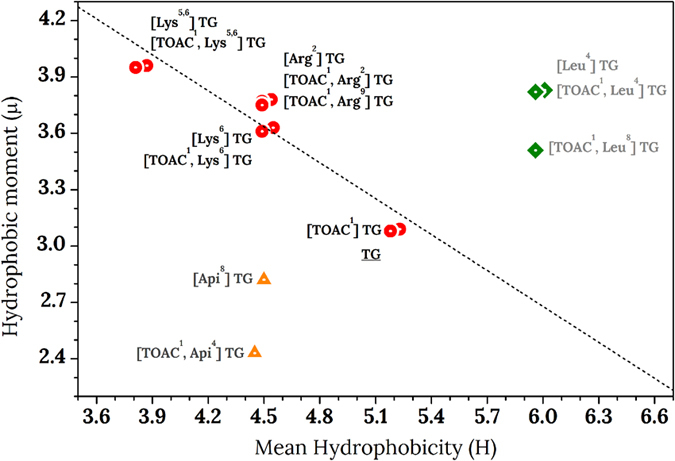
Eisenberg plot for the TG analogues studied in this work. Cytotoxic peptides are represented in red circles; non-toxic peptides in green diamonds and selective peptides in yellow triangles. The dashed line indicates the linear correlation between μ and H of the toxic TG analogues.

The results of the MTT assay (Table 1) make clear that a subtle equilibrium exists between the physico-chemical and structural parameters of the analogues and their cytotoxicity. A measure of this modulation can be obtained from the Eisenberg graph, where H *versus* μ are plotted for all the analogs (Fig. 6). This plot has been created in the mid-80s to categorize proteins depending on their surface-seeking propensity^19^,^38^, and has been more recently used to seek a correlation between H, μ and peptide activity. Unfortunately, due to the complex interplay between the many structural and chemical parameters that govern peptides behaviour, cytotoxic peptides can be hardly distinguished from inactive ones only on the basis of their plot localization^21^,^42^. Conversely, for our TG analogues, the plot clearly shows that there is a good linear correlation (R = 0.94) between H and μ for all the cytotoxic peptides, while the other peptides fall in other regions of the plot. It is interesting to note that the peptides carrying the Aib-to-Api substitutions are out from the linear correlation of the plot and fall in a region that is diametrically opposed to that occupied by the inactive Aib-to-Leu substituted analogues. This observation suggests that only particular pairs of H and μ values result in active peptides: this peculiarity may provide an explanation to the unexpected cytotoxicity displayed by Leu-bearing and Api-bearing TG analogs. In particular, substitutions that decrease the hydrophobic moment, perturbing the hydrophilic-hydrophobic balance, induce a selective toxicity towards a specific cell line. It may be surprising that analogues with a strongly enhanced hydrophobicity result in activity loss, since substitutions favouring van der Waals interaction enhance the membrane partitioning of these peptide. This result indicated that a high affinity to the membrane, also revealed by EPR measurements (see Table 2), does not forcedly implicate toxicity.

To gain greater insight on the different cytotoxicity of the analogues, we studied their interaction with the cell membranes performing EPR and microscopy experiments to determine the binding and localization of the peptides. The microscopy experiments show that the peptides locate in the cell membrane and kill the cells *via* membrane lysis (Fig. 5), and do not interact with mitochondria. The EPR results show that no correlation exists between the amount of peptide bound to the cell membrane and cytotoxicity, since three of the four analogues show very high binding to the three cell lines irrespective of their toxicity (Table 2). The fourth peptide, [TOAC^1^, Api^4^] TG displays an expected low binding due to the Aib-to-Api substitution that introduces a charge in the hydrophobic face, thus perturbing the interaction of the helix with the hydrocarbon region of the bilayer. Remarkably, [TOAC^1^, Api^4^] TG has a significantly higher affinity for T67 cells, towards which it is selectively toxic. The higher binding to T67 cells than to HeLa and HDF results from the higher negative charge on the outer surface of T67 cells, that favours the binding of the positively charged peptide. These evidences suggest that, although cytotoxicity cannot be directly related to the affinity of a peptide for the membrane surface (which regulates the level of binding), nevertheless, a critical peptide concentration on the membrane surface must be reached to trigger toxicity and membrane lysis.

The plasma membrane is recognized to be inhomogeneous, with regions enriched in particular lipids and/or proteins. The inhomogeneity raises the question whether peptides bind preferentially to regions with a specific composition^3^. For this reason, we determined the amount of cholesterol in the different cell lines (Fig. 2, bottom) since it has been suggested that an abundance of cholesterol might inhibit peptide binding. The reduced binding has been attributed to the presence of regions of reduced fluidity, lipid rafts^43^, commonly thought to be composed of sphingolipids and cholesterol. The exact nature of rafts is elusive: while cholesterol and sphingolipids have been shown to form complexes^44^, recent works suggested that cholesterol is uniformly distributed in the cell membrane^45^. In this context, we found that cholesterol content varies significantly between the cell lines, however it does not correlate either with peptide toxicity or peptide binding. Additionally, molecules localized in regions with different fluidity are expected to show different dynamics (i.e. correlation times τ)^46^,^47^,^48^, however, the τ values determined from the simulations of all EPR spectra are similar, irrespective of the amount of cholesterol. Then, the absence of correlation suggests that TG analogues either bind to fluid regions low in cholesterol or that the variations in fluidity are too small to influence peptide dynamics, toxicity, and binding.

In conclusion, our findings can be summarized as follows: (1) we synthesized two analogues ([TOAC^1^, Api^4^] TG and [Api^8^] TG), with a perturbed structural sector of the amphipathic helix, that display selective cytotoxicity towards a cancer cell line; (2) a defined ratio between H and μ is required to retain toxicity, therefore these parameters must be taken into account in the design of future TG analogues; (3) the activity is turned off when the binding falls below a critical threshold. However, binding is not sufficient for the peptides to be toxic and other factors such as their penetration depth, aggregation and/or internalization need to be considered to fully rationalize their activity.

## Methods

### Peptide synthesis and purification

Peptides were prepared by Solid-Phase Peptide Synthesis (SPPS). The synthetic procedure was similar to previously reported protocols^24^,^26^. Peptides [FITC^11^] TG and [Lys^6^, FITC^11^] TG were synthesized by manual SPPS on a 0.1 mmol scale. 1,2-Diaminoethane-trityl resin (Iris Biotech, Marktredwitz, Germany) (126 mg, loading 0.81 mmol/g). Fmoc-deprotection was achieved by treatment with 20% piperidine solution in DMF (repeated two times, 5 and 10 min, respectively). The coupling steps were generally carried out exploiting HBTU and HOBt as activating agents and in the presence of DIEA, except for those steps involving Aib, TOAC, or Api residues, which were doubled and HATU was employed instead of HBTU/HOBt. All the coupling steps were carried out with three equivalent excess of the activated residue and one-hour long. Finally, the reaction with four equivalents of 1-Octanoic acid, EDC and HOAt in the presence of N-methylmorpholine, produced the 1-Oct N-terminal capping. The 1-hour coupling procedure was repeated twice. Peptide cleavage from the resin was achieved by several treatments with 30% HFIP in dichloromethane, as described in ref. 24. The filtrates were collected and concentrated under a flow of N_2_. The crude peptides TG-NH-CH_2_CH_2_NH_2_ and [Lys^6^(Boc)]TG-NH-CH_2_CH_2_NH_2_ were used without purification to obtain the respective, FITC-bearing, compounds [FITC^11^] TG and [Lys^6^, FITC^11^] TG, by reaction with 2 equivalents of FITC in DMF, in the presence of DIEA. The reaction mixtures were diluted with H_2_O, transferred in separating funnels, and the products extracted with CH_2_Cl_2_. Each organic phase was washed with H_2_O three times, dried over Na_2_SO_4_, filtered, and evaporated to dryness. Yield: 37%, Rf_1_: 0, Rf_2_: 0.7, Rf_3_: 0. HPLC (65–100% B in 20 min, 1.0 mL/min): R_t_ = 8.38 min. [Lys6(Boc), FITC^11^] TG was dissolved in HCl 3 M in methanol and left stirring until quantitative Boc-removal was achieved (2 hours, HPLC). The radical character of TOAC was hampered by the acid treatments needed to deprotect Lys or Arg side chains. In particular, exposure to the 95% TFA solution, used to remove Pbf protecting group, caused a severe loss of the radical moiety. To recover the nitroxide group we employed the procedure described by Nakaie and co-workers^30^. Briefly, an aqueous NH_4_OH solution (1 M) was added dropwise to a solution of the TOAC-containing peptide in methanol until it reached pH 9. The mixture was stirred until quantitative TOAC regeneration was achieved (HPLC). However, after treatment with 95% TFA the yield of the TOAC regeneration was never higher than 70%. The crude peptides were purified by means of a Biotage ISOLERA Prime system.

### CD samples preparation and experiments

Spectrograde MeOH (Acros) and 100 mM SDS were used as solvents. The SUV used in this study were prepared from POPC. The lipids were dissolved in chloroform and a homogeneous lipid film was obtained by drying the solution under a gentle dry nitrogen stream; the film was left overnight under vacuum in a desiccator to remove any trace of solvent. The following day the film was resuspended in HEPES buffer (5 mM, pH 7.4) to obtain a lipid concentration of 2 mM. SUV in the 30–50 nm diameter range were prepared by sonication: the lipid suspension was immersed in a water bath and sonicated until the solution started to clear. Vesicle dimensions were checked during preparation by dynamic light scattering using a NICOMP Model 370 Submicron Particle Sizer by Pacific Scientific. The peptides were incorporated in the liposomes as follows: a methanol solution of the peptide was evaporated in an Eppendorf tube. The pre-formed liposomes were added and the solution was then sonicated for 3 min. Vesicles were used fresh after preparation. CD recordings were carried out on a JASCO J-715 (Tokyo, Japan) spectropolarimeter. The CD spectra were acquired and processed using the J-700 program for Windows. All spectra were recorded at room temperature, using Hellma (Müllheim, Germany) cylindrical quartz cells with Suprasil windows, optical path-lengths of 0.01 cm, a bandwidth of 2 nm, and a time constant of 2 s at a scan speed of 20 nm/min. The signal-to-noise ratio was improved by accumulating 36 scans followed by digital signal processing. The values are expressed in terms of [θ]_R_, the total molar ellipticity [deg∙cm^2^∙dmol^−1^].

### Cell culture

Culture media, foetal bovine serum (FBS) and antibiotics were purchased from Carlo Erba Dasit Group. The T67 human glioma cell line was derived by Lauro *et al.*^49^ from a World Health Organization (WHO) Grade III gemistocytic astrocytoma. Human dermal fibroblasts (HDF) were obtained by ATCC. HDF were cultured in Fibroblast Basal Medium (ATCC) supplemented with 10% FBS. T67 and HeLa cells were cultured in Dulbecco’s modified Eagle’s medium (MMedical) supplemented with 10% FBS, 100 UI/ml penicillin, 100 μg/ml streptomycin, and 40 μg/ml gentamycin, and kept in a 5% CO_2_ atmosphere at 37 °C, with saturating humidity. Cell viability and number were measured by trypan blue exclusion method^50^.

### MTT assays

Cytotoxicity of peptides was estimated using a MTT assay. All the chemicals used for this assay have been purchased by sigma Aldrich and used without further purification. T67, HeLa and HDF cells were seeded in 24-well plates at 1 × 10^5^ cells/well. Experiments were performed after 24 h of incubation at 37 °C in 5% CO_2_. After this time cells were washed and treated for 24 h with different concentrations of peptides; the cells are then incubated for 60 mins with 300 μM of MTT in culture medium. The absorbance of each well was measured at 450 nm with a spectrofluorometer (Wallac Victor multilabel counter; Perkin-Elmer Inc., Boston, MA, USA). Data are reported as the mean ± standard deviation of at least three independent experiments^50^.

### Annexin V assay

The phosphatidylserine (PS) located on the outer leaflet of the plasma membrane was measured using Alexa Fluor^®^ 488 Annexin V Kit (Molecular Probes, Invitrogen) following manufacturer instruction. The fluorescent signal was detected by flow cytometry using a Beckman-Coulter Epics XL-MCL cytofluorimeter at λ_exc_ = 488 nm, λ_em_ = 525 nm.

### Cholesterol quantification

The cholesterol content of cultured cells was determined using the cholesterol oxidase method with minor variations^51^. Pelleted cells were treated with lysis buffer (0.1% Triton X-100 v/v) to a final protein concentration of 0.5 mg/ml. Then, 10 μL of reaction mix (500 mM MgCl_2_, 500 mM Tris buffer (pH 7.4), 10 mM ditiotreitol, 5% sodium cholate (w/v), 0.1% Triton X-100 (w/v), 0.8 U/ml cholesterol oxidase and 0.8 U/ml cholesterol esterase) were added to 100 μL of cell suspension. The solution was incubated at 37 °C for 30 minutes and was stopped by adding 100 μl of a 1:1 methanol/ethanol (v/v) solution and incubated at 0 °C for 30 min. After 10 min of centrifugation at 10000 × g, 10 μL of the supernatant was analysed by HPLC. The oxidized cholesterol was separated on a Kinetex C18 column (100 × 4.6 mm, 2.6 μm; Phenomenex, CA, USA) using a mobile phase consisting in 1:99 acetic acid/methanol (v/v) at a flow rate of 0.4 ml/min with a Agilent 1100 HPLC system. Absorbance at λ = 240 nm was monitored by a photodiode array detector. To quantitate the amount of cholesterol, external cholesterol calibration standards were used.

### EPR samples preparation and measurements

Methanol, 99.9%, spectrophotometric grade, [4-(2-hydroxyethyl)-1-piperazinyl]ethanesulfonic acid (HEPES), and PBS buffer (NaCl 137 mM, KCl 2.7 mM, Na_2_HPO_4_ 10 mM, KH_2_PO_4_ 1.8 mM, pH 7.4) were purchased from Sigma-Aldrich. A 50 mM, pH 7.0, HEPES buffer solution was prepared to be used for liposomes. 1-palmitoyl–2-oleoyl-*sn*-glycero-3-phosphocholine (POPC) was purchased from Avanti Polar Lipids, Alabaster (AL) as chloroform solution. For the EPR experiments in liposomes, POPC liposomes preparation and peptide incorporation were carried out in the same way as for CD experiments; the peptide to lipid ratio was 1:100 and the peptide concentration was 78 μM, identical for peptides in SUV and in buffer. For the EPR experiments in cells, T67, HeLa and HDF cells were cultured as described above and then stored in liquid nitrogen as a 5∙10^6^ cells/mL suspension in PBS buffer until use. The frozen suspension was quickly warmed in a 37 °C water bath and washed twice with PBS. The peptides were incorporated in the cells as described in the CD section; the 3 min sonication was avoided to prevent damages to the cells. The concentration of the peptides in the samples was 125 μM and each samples contains 25 nmol of peptides per million cell, a peptide-to-cell ratio comparable to the concentration of 2 μM in the MTT assay. The spectra were recorded after 15–20 minutes of incubation of the peptide to the cell suspension. EPR experiments with cells are sometimes difficult to perform since the cells antioxidants reduce the nitroxide group: however, in our experimental conditions, the TOAC moiety was consumed slowly (>80% of the spins still present after roughly 2 hours) allowing the acquisition of the spectra without distortions. CW-EPR spectra were performed using a Bruker ER200D spectrometer operating at X-band (~9.5 GHz), equipped with a rectangular cavity, ER4102ST, fitted with a cryostat, and a variable-temperature controller, Bruker ER4111VT. The microwave frequency was measured by a frequency counter, HP5342A. All spectra were obtained using the following parameters: microwave power 2.1 mW; modulation amplitude 0.10 mT; modulation frequency 100 kHz; time constant 41 ms; conversion time 82 ms; scan width 12 or 15 mT; 1024 points; temperature 293 K. The spectra were obtained as the average of 9 scans (cells) or 25 scans (SUV and solution). The spectra were simulated with a program based on the stochastic Liouville equation^52^, that is extensively used to simulate the EPR spectra of spin-labelled peptides, proteins and lipids^53^,^54^,^55^,^56^,^57^, the details are reported in the Supplementary Information.

### Fluorescence microscopy

HeLa cells were washed with PBS (NaCl 137 mM, KCl 2.7 mM, Na_2_HPO_4_ 10 mM, KH_2_PO_4_ 1.8 mM, pH 7.4), detached with 0.05% trypsin–EDTA solution and centrifuged for 3 min at 300 × g. The pellet was resuspended in DMEM medium and cells were counted by using trypan blue exclusion method. Then, 5 × 10^3^ cells were seeded on custom chambers^58^ with glass bottom, in 100 μL of complete DMEM medium and incubated at 37 °C in a humidified atmosphere with 5% CO_2_. After 24 h, cells were washed with PBS and incubated for 3 hours with 10 μM of [FITC^11^] TG and [Lys^6^, FITC^11^] TG dissolved in DMEM. After this time, cells were carefully washed with PBS, then 50 μL of PBS supplemented with calcium and magnesium (NaCl 137 mM, KCl 2.7 mM, Na_2_HPO_4_ 10 mM, KH_2_PO_4_ 1.8 mM, CaCl_2_ 1 mM, MgCl_2_ 0.5 mM, pH 7.4) was added to each chamber. Finally, chambers were mounted on the microscope stage for analysis. Micrographs were acquired on a Zeiss Axiovert 200 inverted microscope equipped with XBO xenon 75 W short arc lamp and a Photometrics Coolsnap FX CCD camera (Roper Scientific) with Metafluor Imaging Software version 6.1r6 (Universal Imaging Inc.) at λ_exc_ = 500 nm, λ_em_ = 545 nm.

## Additional Information

**How to cite this article**: Dalzini, A. *et al.* The rational search for selective anticancer derivatives of the peptide Trichogin GA IV: a multi-technique biophysical approach. *Sci. Rep.* **6**, 24000; doi: 10.1038/srep24000 (2016).

## Supplementary Material

Supplementary Information

## Figures and Tables

**Table 1:** Primary sequence, net charge, hydrophobicity (H), hydrophobic moment (μ), R value (see text), and EC50 values for the peptides studied in this work.

Peptide	Primary sequence^a^	Net charge	H	μ	R = [θ]_222_/[θ]_208_		EC50^b^ (μM)		
					MeOH	SDS	T67	HeLa	HDF
TG	*Oct*-UGLUGGLUGI-Lol	0	5.23	3.09	0.2	0.5	2	8	4
[TOAC^1^] TG	*Oct*-XGLUGGLUGI-Lol	0	5.18	3.08	0.3	0.6	1	3	5
[Lys^6^] TG	*Oct*-UGLUGKLUGI-Lol	+1	4.55	3.63	0.4	0.6	4	2	2
[TOAC^1^, Lys^6^] TG	*Oct*-XGLUGKLUGI-Lol	+1	4.49	3.61	0.5	0.7	1	3	5
[Arg^2^] TG	*Oct*-URLUGGLUGI-Lol	+1	4.54	3.78	0.4	0.6	8	4	8
[TOAC^1^, Arg^2^] TG	*Oct*-XRLUGGLUGI-Lol	+1	4.49	3.77	0.3	0.6	8	9	15
[TOAC^1^, Arg^9^] TG	*Oct*-XGLUGGLURI-Lol	+1	4.49	3.75	0.4	0.6	8	5	8
[Lys^5,6^] TG	*Oct*-UGLUKKLUGI-Lol	+2	3.87	3.96	0.5	0.5	7	10	15
[TOAC^1^, Lys^5,6^] TG	*Oct*-XGLUKKLUGI-Lol	+2	3.81	3.95	0.6	0.8	10	6	15
[TOAC^1^, Api^4^] TG	*Oct*-XGLZGGLUGI-Lol	+1	4.45	2.43	0.5	0.6	8	n.t.	n.t.
[Api^8^] TG	*Oct*-UGLUGGLZGI-Lol	+1	4.50	2.82	0.4	0.4	13	n.t.	n.t.
[Leu^4^] TG	*Oct*-UGLLGGLUGI-Lol	0	6.01	3.83	0.3	0.6	n.t.^*c*^	n.t.	n.t.
[TOAC^1^, Leu^4^] TG	*Oct*-XGLLGGLUGI-Lol	0	5.96	3.82	0.4	0.7	n.t.	n.t.	n.t.
[TOAC^1^, Leu^8^] TG	*Oct*-XGLUGGLLGI-Lol	0	5.96	3.51	0.2	0.7	n.t.	n.t.	n.t.

The top part of the table contains the toxic peptides, the middle part the selective peptides, the bottom part the non-toxic peptides. ^*a*^*Oct*, 1-octanoyl; Lol, leucinol; U, Aib; X, TOAC; Z, Api. ^*b*^The EC50 values have an average error of about ±2 μM. ^*c*^n.t. = not toxic up to 20 μM.

**Table 2:** Isotropic rotational correlation time of membrane-bound peptides (τ) and percentage of bound peptide obtained from the simulations of the EPR spectra.

Peptide	Correlation time, τ				% membrane-bound peptide			
	POPCSUV^a^	T67	HeLa	HDF	POPCSUV^b^	T67	HeLa	HDF
[TOAC^1^] TG	9 ns	9 ns	12 ns	9 ns	100%	85%	78%	84%
[TOAC^1^, Lys^6^] TG	n.a.	10 ns	8 ns	9 ns	100%	96%	89%	97%
[TOAC^1^, Api^4^] TG	n.a.	12 ns	12 ns	12 ns	>99%	41%	21%	28%
[TOAC^1^, Leu^4^] TG	n.a.	8 ns	9 ns	8 ns	100%	97%	96%	95%

^a^all spectra in SUV have the same lineshape, only the one for peptide [TOAC^1^] TG has been simulated. ^b^the binding percentages between SUV and cells are not directly correlated since the concentrations in the two experiments are not comparable. Principal values of g tensor used in the simulations: g_xx_ = 2.0096, g_yy_ = 2.0064, g_zz_ = 2.0027. Principal values of A tensor: A_xx_ = A_yy_ = 0.56 mT. The values of A_zz_ are: 3.75 mT for peptides in buffer, 3.27 mT for peptides in SUV, and 3.35 mT for peptides in cells. The variations of A_zz_ are discussed in text.
