# Supplementary material for: The rational search for selective anticancer derivatives of the peptide Trichogin GA IV: a multi-technique biophysical approach
Source: Sci Rep. 2016 Apr 4;6:24000. doi: 10.1038/srep24000 (PMC4819177; doi:10.1038/srep24000)
Supplement: Supplementary Information [file srep24000-s1.doc]

**Supplementary Information**

**for**

**The rational search for selective anticancer derivatives of the peptide Trichogin GA IV: a multi-technique biophysical approach**

Annalisa Dalzini^†^, Christian Bergamini^‡^, Barbara Biondi^†^, Marta De Zotti^†^, Giacomo Panighel^†^, Romana Fato^‡^, Cristina Peggion^†^, Marco Bortolus^†§*^, and Anna Lisa Maniero^†*^

^†^Dipartimento di Chimica, Università di Padova, via Marzolo 1, 35131, Padova, Italy.

^‡^Dipartimento di Farmacia e Biotecnologie, Università di Bologna, via Irnerio 48, 40126, Bologna, Italy.

^§^Dipartimento di Scienza dei Materiali, Università degli Studi di Milano Bicocca, 20126, Milano, Italy.

***Details of simulation of EPR spectra***

The spectra of the TOAC-labeled peptides were simulated with a program based on the stochastic Liouville equation [Budil, D. E., Lee, S., Saxena, S., and Freed, J. H. (1996) *Nonlinear-least-squares analysis of slow-motion EPR spectra in one and two dimensions using a modified Levenberg-Marquardt algorithm*, Journal of Magnetic Resonance, Series A 120, 155-189]. The simulation method relies on several reference systems: the g and ^14^N hyperfine (A) tensors of the TOAC label are considered collinear and their orientation relative to the rotational diffusion reference frame is determined by a set of Euler angles. However, in this work, we simplified the simulation approach using a single isotropic value (D_iso_) for the diffusion tensor. The rotational correlation times τ reported in the work are related to the diffusion tensor via the classic equation:
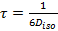
.

We performed a global fitting of the spectra letting both the D_iso_, g and hyperfine tensors free to float and found that the only parameters that affected significantly the spectral simulations were A_zz_ and D_iso_. Then, the principal values of the g (g_xx_=2.0096, g_yy_=2.0064, g_zz_=2.0027) and A (A_xx_ = A_yy_ = 0.56 mT) tensors were obtained from the literature [Inbaraj, J. J., Cardon, T. B., Laryukhin, M., Grosser, S. M., and Lorigan, G. A. (2006) *Determining the topology of integral membrane peptides using EPR spectroscopy*, Journal of the American Chemical Society 128, 9549-9554], except for the value of the A_zz_ component. Since the values of the A_zz_ hyperfine component depend strongly on the polarity of the environment, its value has been optimized: in all environments, while A_zz_ = 3.27 mT for peptides bound to SUV, A_zz_ = 3.35 mT for peptides bound to cells and A_zz_ = 3.75 mT for peptides in solutions.

***Full EPR spectra of TG analogs in SUV and cells.***

In Figure S1, the EPR spectra of all TOAC-labeled TG analogs recorded in SUV are shown. The EPR spectra of [TOAC^1^, Api^4^] TG and [TOAC^1^, Arg^9^] TG show additional contributions indicative of the presence of peptides in aqueous solution (less than 1% of the total spins). For clarity, in the top part of Figure S1, we report the spectrum of [TOAC^1^] TG in buffer: the spectrum shows only the three sharp lines typical of a fast-moving unbound peptide in aqueous solution.


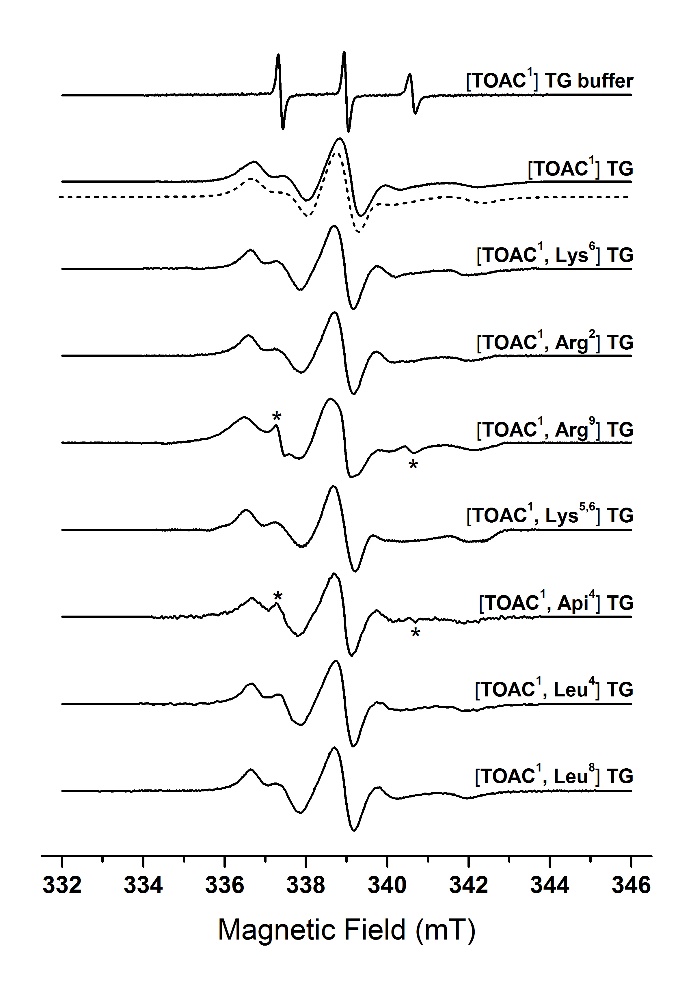


**Figure S1** EPR spectra of the TOAC-labeled TG analogs (see Table 1) interacting with 10 mM POPC SUV and, in the top part, of [TOAC^1^] TG in HEPES buffer (pH=7.0, no salts). In dashed line the simulation of the spectrum of [TOAC^1^] TG. P:L ratio 1:100, room temperature. All spectra have been normalized to unit intensity for convenience. The asterisks indicate the spectral contributions of the small fraction of peptides in solution.

In Figure S2, we show the EPR spectra at two P:L ratios (1:250 and 1:100) of selected analogs: the spectra show no significant differences suggesting that at the two P:L ratios no aggregation or changes in the bilayer take place. Please note that the temperature changes from room temperature (about 293 K) to 308 K from Figure S1 to Figure S2.


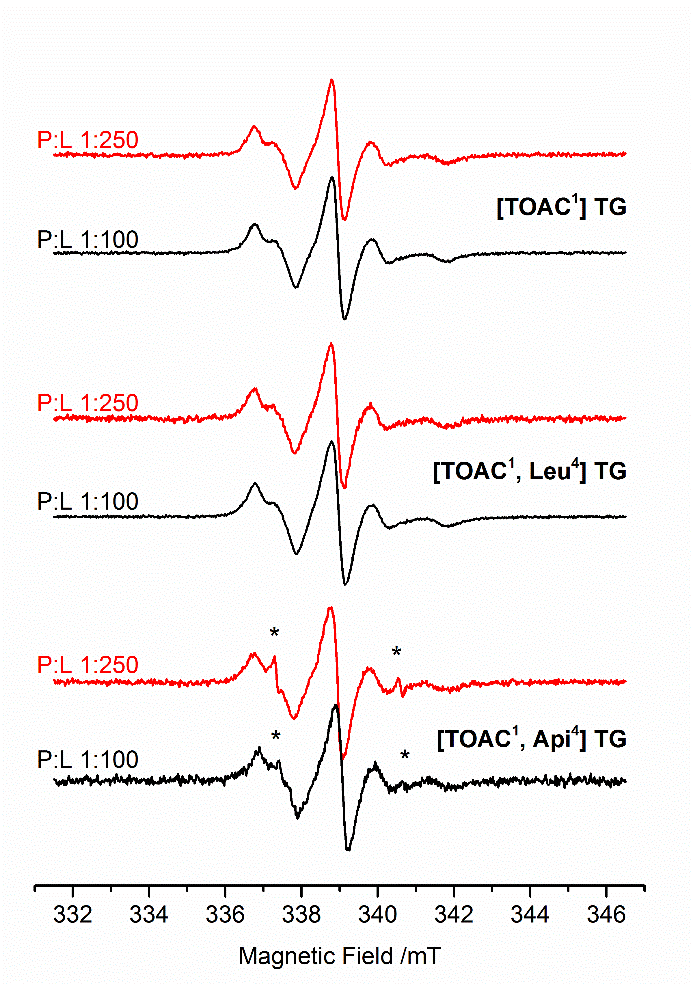


**Figure S2** EPR spectra of selected TOAC-labeled TG analogs (see Table 1) interacting with 10 mM POPC SUV. In black lines the spectra at a P:L ratio 1:100; in red lines the spectra at a P:L ratio 1:250, T = 308 K. All spectra have been normalized to unit intensity for convenience. The asterisks indicate the spectral contributions of the small fraction of peptides in solution.

In Figure S3, the EPR spectra of selected TG analogs recorded in cells at room temperature (293 K) are shown.


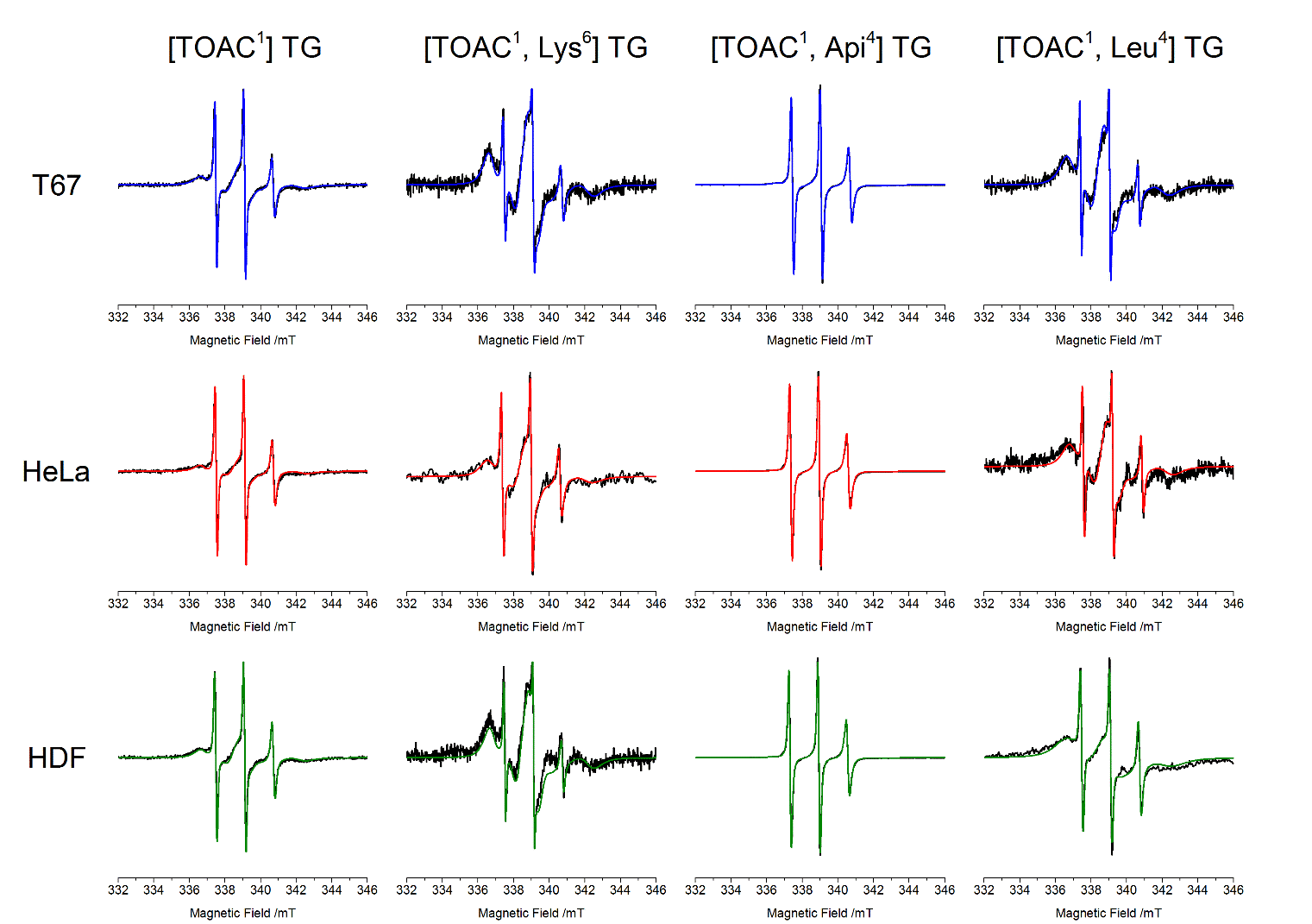
**Figure S3** EPR spectra of selected TOAC-labeled TG analogs interacting with the different cell lines. Experimental spectra in black, simulations in blue, red and green lines for T67, HeLa, and HDF respectively. From left to right the different analogs; from top to bottom the different cell lines. The percentage of bound peptides are reported in Table 2. Temperature 293 K, peptide to cell ratio: 25 nmol/10^6^ cells. All spectra have been normalized to unit intensity for clarity.

***Analysis of the EPR Spectra of TG Analogs in Cells***

In the four panels of Figure S4, we show the EPR spectra of four analogs (the same chosen for the CD experiments in SUV) interacting with T67 cells. The complete simulation of each spectrum is shown in blue and the individual components in red (peptide free in solution) and in green (peptide bound to the membrane). The relative amounts of peptide in the two phases are obtained from the double integral of the simulations of the individual components, i.e. the area under the first integral of the simulations shown in the right side of each panel.


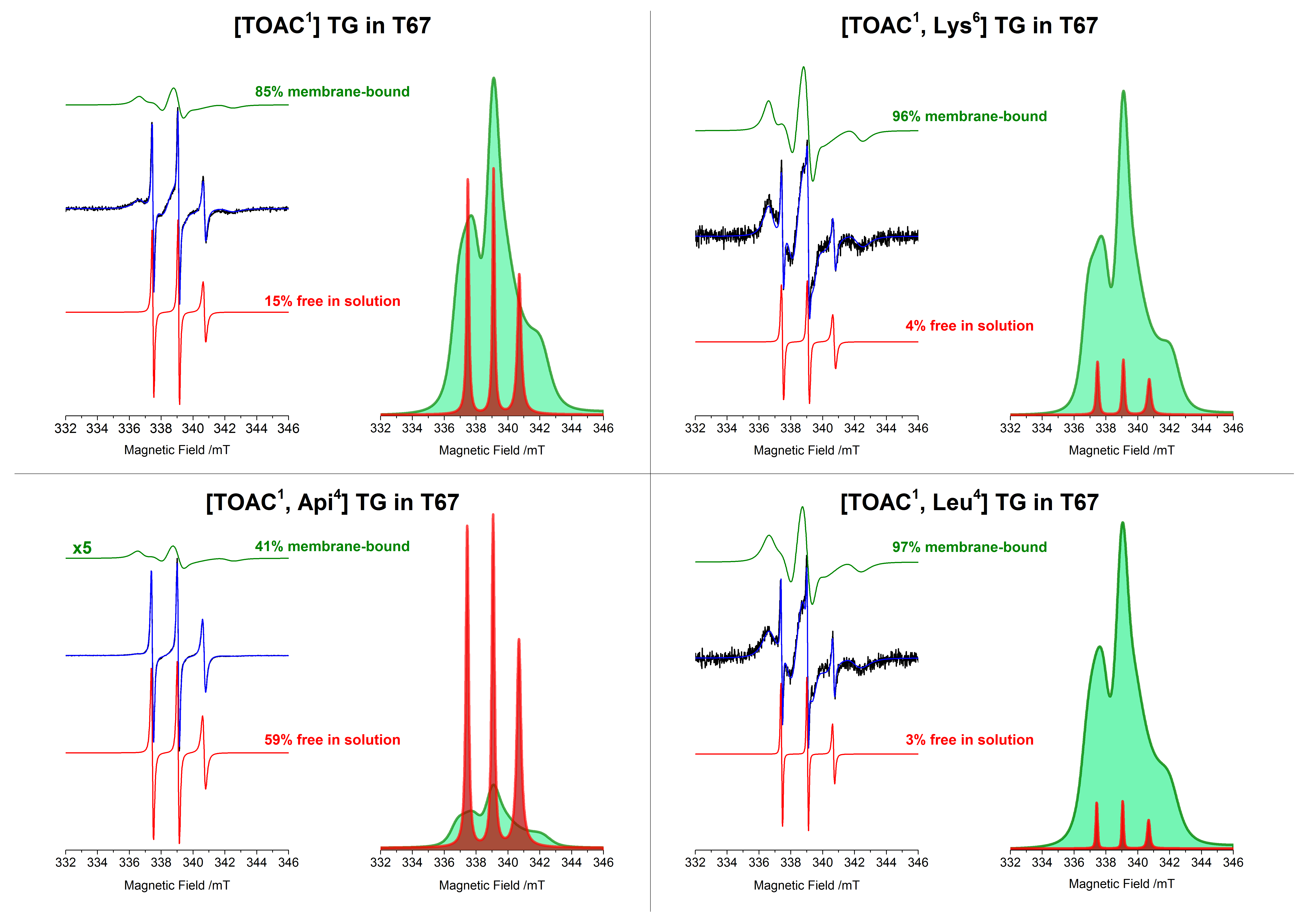


**Figure S4** EPR spectra and simulations of [TOAC^1^] TG, [TOAC^1^, Lys^6^] TG, [TOAC^1^, Api^4^] TG and [TOAC^1^, Leu^4^] TG interacting with T67 cells. On the left side of each panel, the experimental spectra and simulations; on the right side, the first integral of the individual fits with the relative percentages obtained from the areas. Experimental spectra in black; global fits in blue; fits of the membrane-bound peptides in green; fits of the peptides free in solution in red.

***Fluorescence Microscopy***

We performed fluorescence microscopy experiments with rhodamine, which selectively stains mitochondria, to exclude that TG analogs penetrate inside the mitochondria and are localized only on the plasmatic membrane. Rhodamine staining is shown in FigureS5.B in red. The merged images of the experiments on [FITC^11^] TG are shown in Figure S5.C. As can be seen, [FITC^11^] TG keeps the cells intact and resides only on the outside of the cells and no significant colocalization can be observed.


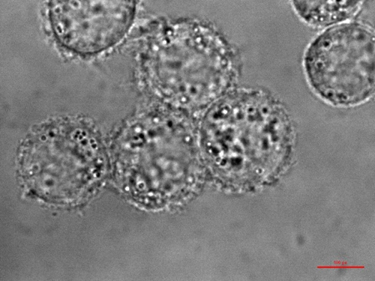

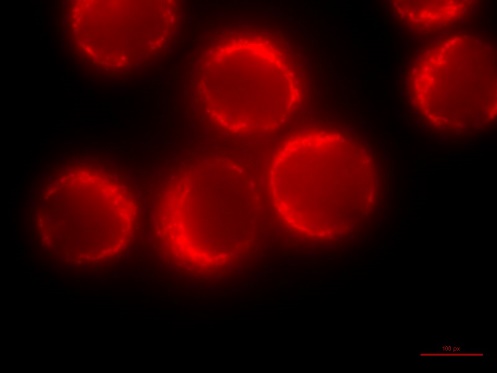

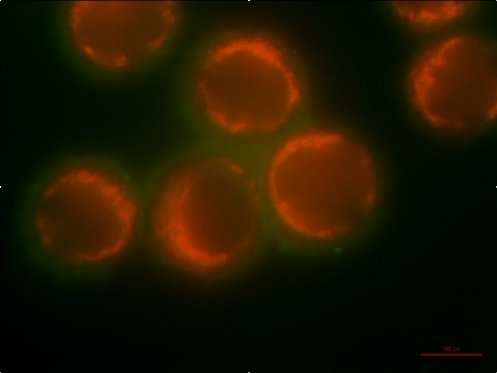


**A**

**B**

**C**

**Figure S5**. Fluorescence microscopy of HeLa cells treated with 0.5 μM of TG-FITC conjugate analogs for 3 hours and then stained with rhodamine. (A) Bright field images in the presence of [FITC^11^] TG; (B) red fluorescence images (rhodamine); (C) the merging of red and green fluorescence fields.

***Validation of the Calculated Mean Hydrophobicity***

The validity of the calculated hydrophobicity values has been confirmed by comparison with the retention times in reverse phase HPLC of a selection of peptides with different hydrophobicity using the following conditions. The calculated values of the mean hydrophobicity are plotted against the retention times in Figure S6.


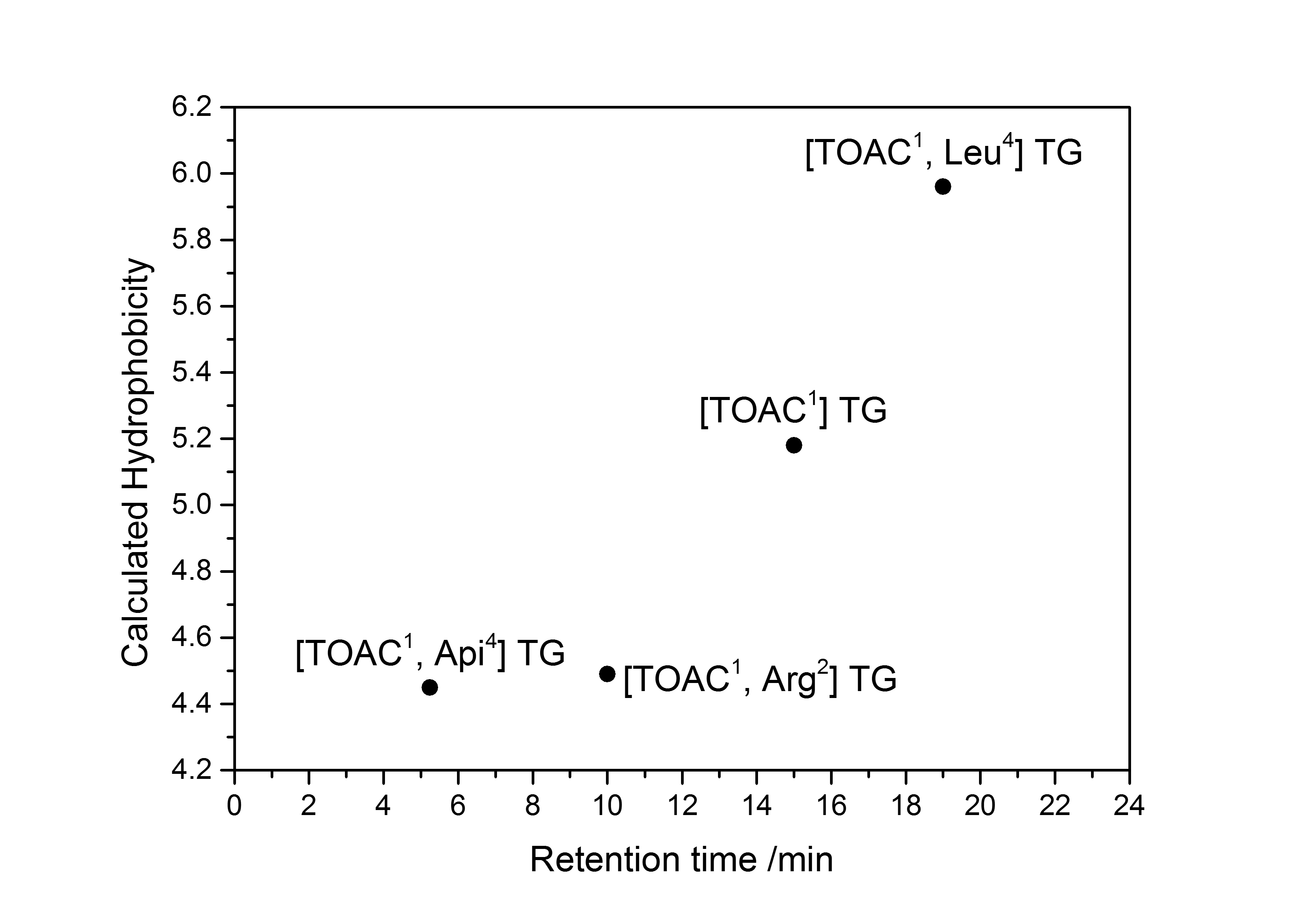


**Figure S6**. Calculated H of selected peptides plotted against their retention times on reverse phase HPLC. Eluant system: solvent A, H_2_O/CH_3_CN 9:1 + 0.05% TFA; solvent B, CH_3_CN/H_2_O 9:1 + 0.05% TFA; gradient 60-100% B in 30 mins; Phenomenex C18 Jupiter 300A column.

***Dynamic Light Scattering***

The dimension of the SUV prepared by sonication was verified by dynamic light scattering (DLS). Additionally we checked if the peptides at a P:L ratio 1:100, the ratio used for EPR experiments, alter the dimension of the SUV. The experiments were perfomed on POPC SUV (2 mM) with and without peptides. We chose a toxic peptide, [Arg^2^] TG, and a non-toxic peptide, [Leu^4^] TG. The DLS data by volume and by number are reported in Table S1. The average diameter of the SUV in this preparation is about 21±6 nm (when counting the size distribution by numer). As can be seen, the addition of the peptides does not alter the dimension of the vesicles in a significant way.

**Table S1** DLS data for the SUV prepared by sonication used in this work.

|  | Distribution by Volume | | | Distribution by Number | | |
| --- | --- | --- | --- | --- | --- | --- |
|  | Peak 1 | Peak 2 | Peak 3 | Peak 1 | Peak 2 | Peak 3 |
| SUV | 31 nm (97%) | 5147 nm (3%) | - | 21 nm (100%) | - | - |
| SUV + [Arg^2^] TG | 28 nm (92%) | 5430 nm (3%) | 135 nm (5%) | 24 nm (100%) | - | - |
| SUV + [Leu^4^] TG | 30 nm (96%) | 4993 nm (4%) | - | 20 nm (100%) | - | - |
